# Plasmodesmal endoplasmic reticulum proteins regulate intercellular trafficking of cucumber mosaic virus in Arabidopsis

**DOI:** 10.1093/jxb/erad190

**Published:** 2023-05-21

**Authors:** Byung-Kook Ham, Xiaohua Wang, Roberto Toscano-Morales, Jinxing Lin, William J Lucas

**Affiliations:** Global Institute for Food Security (GIFS), University of Saskatchewan, 421 Downey Rd, Saskatoon, SK S7N 4L8, Canada; Department of Biology, University of Saskatchewan, 112 Science Place, Saskatoon, SK S7N 5E2, Canada; Department of Plant Biology, College of Biological Sciences, University of California, Davis, CA 95616, USA; Key Laboratory of Plant Resources, Institute of Botany, Chinese Academy of Sciences, Beijing 100093, China; Department of Plant Biology, College of Biological Sciences, University of California, Davis, CA 95616, USA; Key Laboratory of Plant Resources, Institute of Botany, Chinese Academy of Sciences, Beijing 100093, China; Department of Plant Biology, College of Biological Sciences, University of California, Davis, CA 95616, USA; University of Maryland, USA

**Keywords:** Cucumber mosaic virus, ER proteins, non-cell autonomous protein, plasmodesmata, viral movement protein, virus infection

## Abstract

Plasmodesmata (PD) are plasma membrane-lined cytoplasmic nanochannels that mediate cell-to-cell communication across the cell wall. A range of proteins are embedded in the PD plasma membrane and endoplasmic reticulum (ER), and function in regulating PD-mediated symplasmic trafficking. However, knowledge of the nature and function of the ER-embedded proteins in the intercellular movement of non-cell-autonomous proteins is limited. Here, we report the functional characterization of two ER luminal proteins, AtBiP1/2, and two ER integral membrane proteins, AtERdj2A/B, which are located within the PD. These PD proteins were identified as interacting proteins with cucumber mosaic virus (CMV) movement protein (MP) in co-immunoprecipitation studies using an Arabidopsis-derived plasmodesmal-enriched cell wall protein preparation (PECP). The AtBiP1/2 PD location was confirmed by TEM-based immunolocalization, and their AtBiP1/2 signal peptides (SPs) function in PD targeting. *In vitro*/*in vivo* pull-down assays revealed the association between AtBiP1/2 and CMV MP, mediated by AtERdj2A, through the formation of an AtBiP1/2–AtERdj2–CMV MP complex within PD. The role of this complex in CMV infection was established, as systemic infection was retarded in *bip1/bip2w* and *erdj2b* mutants. Our findings provide a model for a mechanism by which the CMV MP mediates cell-to-cell trafficking of its viral ribonucleoprotein complex.

## Teaser

Plant viruses move through plasmodesmata to traffic between tissues in host plants. We identified plasmodesmata-localized ER proteins which function in control over the spread of cucumber mosaic virus in Arabidopsis.

## Introduction

In plant cells, plasmodesmata (PD) are intercellular nanochannels that establish cytoplasmic continuity between adjacent cells to allow symplasmic delivery of a wide range of molecules, including metabolites, hormones, proteins, and diverse RNA species, which function in plant growth and development, and responses to abiotic and biotic challenges (Lucas *et al.*, 2009; Ham and Lucas, 2014; Kehr and Kragler, 2018; Lu *et al.*, 2018; Mellor *et al.*, 2020; Kehr *et al.*, 2022; Mehra *et al.*, 2022). The cytoplasmic sleeve within the PD, which is the space between the plasma membrane and the endoplasmic reticulum (ER), functions as the main route for molecular trafficking (Zambryski and Crawford, 2000; Zambryski 2008). The PD pore aperture determines permeability to restrict movement of metabolites and macromolecules through PD [referred to as the size exclusion limit (SEL)] and is under dynamic control via molecular processes, including callose deposition/degradation at the PD orifice (Lucas *et al.*, 2009; Amsbury *et al.*, 2017; Tilsner *et al.*, 2018).

Plant viruses can commandeer the endogenous capacity of PD to traffic protein–RNA complexes to infect the tissues of their host plants. Extensive studies conducted with a wide range of plant-infecting viruses, including tobacco mosaic virus (TMV), cucumber mosaic virus (CMV), and potato virus X (PVX), established that their viral-encoded movement proteins (MPs) can both bind their genomic RNAs/DNAs and dilate the PD pore to allow cell-to-cell trafficking of the viral genome (Wolf *et al.*, 1989; Fujiwara *et al.*, 1993; Noueiry *et al.*, 1994; Ju *et al.*, 2005; Lucas, 2006; Sasaki *et al.*, 2006; Brandner *et al.*, 2008; Su *et al.*, 2010; Pena and Heinlein, 2012;  Yuan *et al.*, 2016). Thus, viral MPs provide a powerful tool to explore the molecular mechanisms by which PD function in mediating cell-to-cell trafficking of macromolecules (Benitez-Alfonso *et al*., 2010).

In this study, we characterized Arabidopsis ER-associated proteins, AtBiP1/2 and AtERdj2A/B, which locate within PD and function in CMV spread. AtBiP1/2 are ER-luminal members of the heat shock protein 70 (HSP70) family and function as molecular chaperons to regulate protein translocation, protein folding, and quality control during ER lumen stress (Nishikawa *et al.*, 2005; Wakasa *et al.*, 2011). AtERdj2A/B are members of the ER-localized DnaJ family and have been characterized as BiP partners for protein translocation across the ER membrane (Yamamoto *et al.*, 2008; Pobre *et al.*, 2019). AtBiP1/2 and AtERdj2A/B were co-immunoprecipitated with the CMV MP and our *in vitro*/*in vivo* pull-down studies established that the interaction between CMV MP and AtBiP1/2 is mediated by AtERdj2A, through the formation of an AtBiP1/2–AtERdj2–CMV MP complex within the PD. The role of this complex in CMV infection was established, as systemic infection was retarded in *bip1/bip2w* and *erdj2b* mutant plants. Our findings provide a model for the mechanism by which the CMV MP mediates cell-to-cell trafficking of its viral ribonucleoprotein complex, via a novel ER-mediated PD pathway.

## Materials and methods

### Plant materials and growth conditions

Wild-type (Col-0), transgenic and mutant *Arabidopsis thaliana* plants were grown in controlled-environment chambers under long-day conditions (16 h light/8 h dark cycle; 22 °C constant temperature; 200 μmol m^−2^ s^−1^ photosynthetically active radiation). *Nicotiana benthamiana* and *Cucurbita maxima* plants were grown as described previously (Taoka *et al.*, 2007). All T-DNA knockout mutant lines [CS856879 (*bip1-4*) for AT5G28540; CS842467 (*bip2-1*) and SALK_047956 (*bip2-2*) for AT5G42020; SALK_024133 (*bip3-1*) for AT1G09080; CS846905 (*erdj2a-1*) for At1g79940; SALK_007095 (*erdj2b-1*) and SALK_117783 (*erdj2b-2*) for At4g21180; and SALK_103280 (*erdj3a-1*) for At3g08970] were obtained from the ABRC. The *bip1/bip2w* double mutant was obtained by crossing between the *bip1-4* and *bip2-2* mutants. PCR analysis was employed to confirm genotypes, using the appropriate primer sets (Supplementary Table S1).

### Plasmid construction

To purify 4Myc-8His (4M8H)-tagged recombinant proteins, *in planta*, green fluorescent protein (GFP) and CMV MP genes were amplified by PCR and digested with *Sph*I and *Kpn*I to subclone into a zucchini yellow mosaic virus (ZYMV)-based viral vector, as described previously (Ham *et al.*, 2014; Yan *et al.*, 2020). The coding sequence (CDS) of GFP was subcloned as a translational fusion between the N-terminal signal sequence and the rest of the AtBiP1, AtBiP2, and AtBiP3 CDS using overlap-PCR (Supplementary Table S1). All fragments were amplified separately, and subcloned into the pGEM T-easy vector (Promega). The three *AtBiP* signal peptide (SP) genes, *CMV MP*, *AtERdj2A*, along with the *DnaJ* and *CTD* (C-terminal domain) of *AtERdj2A* and *AtERdj3A* were amplified and fused to 4M8H, glutathione *S*-transferase (GST), GFP, and mCherry as described previously (Yan *et al.*, 2020). All constructs were confirmed by sequencing and transformed into *Agrobacterium tumefaciens* strain C58C1.

### PECP preparation and recombinant protein purification

An Arabidopsis plasmodesmata enhanced cell wall protein preparation (PECP) was obtained, as previously described (Lee *et al.*, 2003; Ham *et al.*, 2012). Briefly, a pre-cooled Bead-Beater homogenizer (BioSpeck Products), with 0.5 mm glass beads, homogenization buffer and proteinase inhibitor, was used to homogenize 100 g of Arabidopsis seedlings. Homogenate was precipitated by centrifuging at 300 *g* for 5 min. The precipitated pellet was then resuspended, followed by five additional rounds of centrifugation. The resultant pellet was resuspended with PECP extraction buffer and incubated overnight at 4 °C. The PECP fraction was prepared at 4 °C and stored at −80 °C.

*In planta* recombinant proteins GFP–4M8H and CMV MP–4M8H were transiently expressed in pumpkin plants (*C. maxima*) using the ZYMV viral vector system (Ma *et al.*, 2010; Ham *et al.*, 2012). Briefly, a Helios gene gun system (Bio-Rad) was used to bombard gold particles (1 µm), coated with *GFP* and *CMV MP* constructs in ZYMV vectors, onto pumpkin cotyledons. *CMV MP-GST* and *AtBiP1-GFP-4M8H* constructs were agroinfiltrated into *Nicotiana benthamiana* leaves. A HisTrap FF Column (Cytiva) and a c-Myc-tagged protein mild purification kit (MBL international) were employed to purify 4M8H-tagged recombinant proteins, as described previously (Ham *et al.*, 2012). CMV MP–GST was purified using glutathione–Sepharose 4B-based affinity chromatography (Cytiva).

### Phylogenetic analysis

AtHsp70 and AtERdj2 sequences were collected from the Arabidopsis Information Resource Database (https://www.arabidopsis.org/) and used for sequence alignment. MEGA 11 software was used to construct an AtHsp70 Neighbor–Joining tree in which the consensus tree was generated from 1000 bootstrap replicates.

### Confocal microscopy imaging

The GFP- or mCherry-tagged AtBiP1, AtBiP2, AtBiP3, CMV MP, AtBiP1SP, AtBiP2AP, AtBiP3SP, AtBiP1ΔSP, AtBiP2ΔSP, AtERdj2A, and AtERdj3A were transiently expressed in *N. benthamiana* leaves using agroinfiltration, as described previously (Ham *et al.*, 2012). Zeiss LSM510, Leica TCS SP5, and Leica Stellaris 5 were used for confocal imaging. GFP-tagged proteins were excited with 488 nm and emission was detected at 505–545 nm. The excitation and emission spectra of mCherry-tagged proteins were 561 nm and 587–624 nm, respectively.

### Immunogold labeling for transmission electron microscopy

For high-pressure freezing, root tips were excised from the wild-type Arabidopsis and overexpression lines, and briefly washed in Murashige and Skoog medium. Following a subsequent washing step in hexadecene, root tips were immediately frozen in a high-pressure freezer (HPF010; Bal-Tec). Freeze substitutions were performed in an AFS freeze substitution unit (Leica) in dry acetone supplemented with 0.1% uranyl acetate at −85 ℃ for 3 d before slowly being warmed to −35 ℃ for a period of 18 h. Samples were embedded in Lowycryl HM20, using gelatin capsules. The resin was polymerized with constant UV light for 2 d at −35 ℃ and an additional 3 d at 22 ℃. Thin sections were incubated with anti-BiP2 (Agrisera) and anti-GFP antiserum at a primary dilution of 1:200, followed by incubation with 10 nm gold-coupled secondary antibodies (Biocell GAR10 1:50), at a dilution of 1:30. Aqueous uranyl acetate/lead citrate post-stained sections were examined in a Philips CM10 transmission electron microscope operating at 80 kV.

### Co-immunoprecipitation, *in vitro*/*in vivo* pull-down assays, and immunoblotting analyses

Co-immunoprecipitation (co-IP) experiments were conducted, as described previously (Taoka *et al.*, 2007; Ham *et al.*, 2009). Briefly, an Arabidopsis PECP (500 µg) was dialyzed with binding/wash buffer (0.14 M NaCl, 8.0 mM sodium phosphate, 2.0 mM potassium phosphate, and 10 mM KCl, pH 7.4) overnight, followed by incubation with purified recombinant CMV MP–4M8H (2 µg) for 4 h at 4 °C. Immobilized anti-c-Myc IgG (Thermo Fisher) was added to the Arabidopsis PECP+CMV MP–4M8H mixture and then incubated for 2 h at 4 °C. Immunoprecipitation was conducted using the ProFound c-Myc tag immunoprecipitation/co-IP application set (Thermo Fisher), following the manufacturer’s instructions. SDS–PAGE (12%) was used to separate elution fractions, and gels were then visualized with Gelcode Blue Stain reagent (Thermo Fisher). Excised protein bands were processed and analyzed using LC-MS/MS analysis, as described previously (Ham *et al.*, 2009). The LC-MS/MS datasets were analyzed by using Mascot and X!Tandem against the Arabidopsis database.

The *in vitro* pull-down assays were performed as described previously (Taoka *et al.*, 2007; Ham *et al.*, 2009). Briefly, purified *in planta* recombinant CMV MP–GST (500 ng) and AtBiP–GFP (500 ng) were incubated with binding buffer (0.14 M NaCl, 8.0 mM sodium phosphate, 2.0 mM potassium phosphate, and 11 mM KCl, pH 7.4) for 2 h at 4 ℃. Target proteins were pulled downed using the glutathione–Sepharose 4B-based affinity chromatography kit (Cytiva), followed by immunoblotting analysis with anti-GFP antibody (Ab). For *in vivo* pull-down assays, *AtBiP1-GFP*, *AtERdj2A-4M8H*, *AtERdj2A-DnaJ-4M8H*, *AtERdj2A-CTD-4M8H*, *AtERdj3A-4M8H*, and *CMV MP-GST* constructs were transformed into *A. tumefaciens* (C58C1) and agroinfiltrated into *N. benthamiana* leaves. Total proteins were extracted 4–5 d post-agro-infiltration using extraction buffer [10% glycerol, 25 mM Tris, pH 7.5, 1 mM EDTA, 150 mM NaCl with 10 mM DTT, and 1× protease inhibitor cocktail (Thermo Scientific)]. The c-Myc affinity beads (MBL international) were incubated with extracted total protein (100 µg) at 4 ℃ for 4 h. After three rounds of washing with binding buffer, the samples were eluted with elution buffer (50 mM NaOH), followed by immunoblotting analysis with an anti-c-Myc monoclonal antibody (mAb).

Immunoblotting analyses were performed as described previously (Ham *et al.*, 2012). Briefly, proteins were separated by 12% SDS–PAGE and then transferred onto a nitrocellulose membrane. Immunoblotting analyses used the primary Abs against c-Myc (1:10 000, Sigma-Aldrich), GFP (1:5000, Abcam), and GST (1:2000, Sigma-Aldrich) along with horseradish peroxidase (HRP)-conjugated goat anti-mouse (1:20 000, Sigma‐Aldrich) or HRP-conjugated goat anti-rabbit (1:20 000, Sigma‐Aldrich). The membrane blots were detected using a chemiluminescence reagent (Perkin‐Elmer Life Sciences) and imaged under a GelDoc system (BioRad).

### Viral infection assays

CMV infection assay was performed as described previously (Li *et al.*, 2002; Yuan *et al.*, 2011; Zhang *et al.*, 2017). Briefly, CMV infectious constructs were transformed into *A. tumefaciens* strain EHA105. Agrobacteria were precipitated at 2000 *g* for 15 min, then resuspended in agroinfiltration buffer (10 mM MgCl_2_, 10 mM MES, pH 5.2, and 0.1 mM acetosyringone) to an OD_600_ of 0.7 followed by incubation at room temperature for 3 h. Agrobacteria with CMV infectious constructs were infiltrated into the fifth leaf of Arabidopsis wild-type and mutant plants, termed the inoculated leaf, using a 1 ml syringe. The inoculated leaf and 13th leaf (called systemic leaf 1) were harvested at 7 days after agroinfiltration (dai), and a second cauline leaf (called systemic leaf 2) at 14 dai. CMV infectivity was examined by quantitative real-time PCR (qRT-PCR).

### CMV MP trafficking assays

To examine the cell-to-cell movement of CMV MP–GFP, biolistic particle bombardment was employed to transiently express GFP-fused CMV MP in Arabidopsis leaves (Ueki *et al.*, 2009). Briefly, the CMV MP–GFP construct was coated onto 0.6 µm gold particles (Bio-Rad). Leaves were excised from 5-week-old Arabidopsis plants and bombarded on the abaxial side with DNA-coated gold particles using a Bio-Rad PDS-1000/He Biolistic Particle Delivery System. Fluorescent cells with a typical puncta pattern of CMV MP–GFP were counted at 38–48 h after particle bombardment.

### qRT-PCR

The TRIzol® Reagent (Invitrogen, Life Technologies) was used to extract total RNA, following the manufacturer’s instructions. The cDNA was synthesized from total RNA of 1 µg using the SuperScript IV first-strand synthesis system (Invitrogen). The qRT-PCR analyses were performed with QuantStudio™ 6 and 7 Flex Real-Time PCR Systems (Life Technologies) to detect coat protein (CP) RNA of CMV Fny- and Q-strains, using designed primer sets (Supplementary Table S1). PowerUp™ SYBR™ Green Master Mix (Thermo Fisher) was used for qRT-PCR, and the thermal cycling conditions included initial denaturation at 95 °C for 20 s and 45 cycles of 95 °C for 1 s and 60 °C for 20 s. *AtActin*, translation elongation factor 1α (*AtEF1α*), and poly-ubiquitin (*AtUBQ1*) were used as reference genes to normalize transcript levels. The 2^−∆Ct^ or the 2^−∆∆Ct^ method was employed to calculate transcript levels and ratios, respectively. The Student’s *t*-test was employed for statistical analyses.

## Results

### AtBiP1 interacts with the CMV MP at the PD

**Fig. 1.**
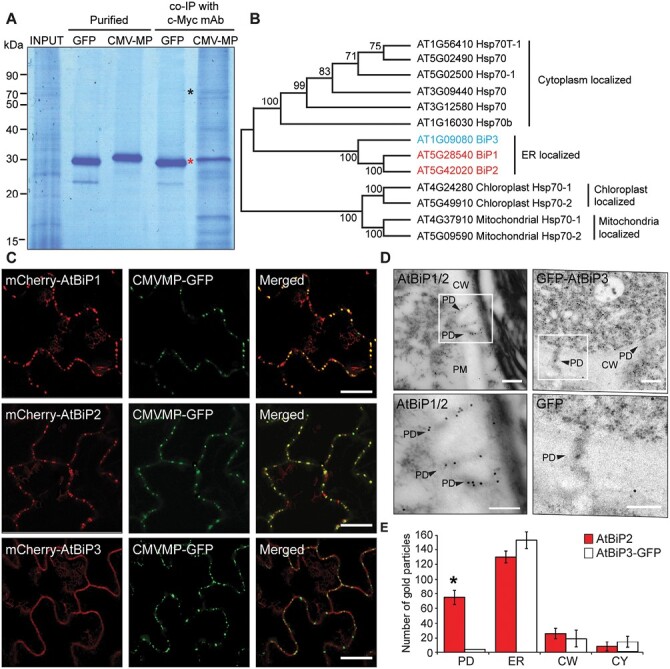
Arabidopsis BiP1 and BiP2, but not BiP3, locate within PD. (A) Identification of CMV MP-interacting proteins in Arabidopsis PECP. The purified recombinant CMV MP or GFP was incubated with Arabidopsis PECP (Input), followed by co-IP using an anti-c-Myc monoclonal antibody (mAb). Red and black asterisks indicate CMV MP and AtBiP1/2, respectively. (B) Phylogenetic analysis of the HSP70 family members using the Neighbor–Joining (NJ) method. The bootstrap value with 1000 replicates is indicated on each branch point. The subcellular localization is described on each clade. (C) AtBiP1/2, but not AtBiP3, co-localized with CMV MP. The N-terminal mCherry-tagged AtBiP1, AtBiP2, or AtBiP3 was co-agroinfiltrated with CMV MP–GFP into *N. benthamiana*. Yellowish signals in merged images represent co-localization of CMV MP–GFP with mCherry–AtBiP1 or mCherry–AtBiP2. Note that *N. benthamiana* epidermal cells were imaged as a single scan plane, using a confocal laser scanning microscope. Scale bar=10 µm. (D) AtBiP1/2 target to PD. Root tissues of Arabidopsis wild-type and GFP–AtBiP3-expressing plants were used for TEM-based immunogold labeling with anti-BiP2 and GFP antibodies, respectively. Lower panels show the magnified images of the boxed regions in the upper panels. Arrowheads indicate gold particles in PD. CW, cell wall. Scale bar=200 nm for upper panels, 50 nm for lower panels. (E) Quantification of AtBiP2 and AtBiP3 immunogold signals in all tested sections. More than 50 individual sections, collected from root samples, were examined to establish the pattern of immunogold labeling. PD, plasmodesmata; ER, endoplasmic reticulum; CW, cell wall; CY, cytoplasm. An asterisk above the error bar indicates significant differences in the number of gold particles that detected AtBiP2 and AtBiP3–GFP within PD, *P*<0.002 based on Student’s *t*-test.

To identify PD proteins that function in the intercellular movement of CMV, co-IP studies were performed on an Arabidopsis PECP using the CMV MP as bait. Of the interacting proteins identified from these CMV MP–PECP co-IP assays, we selected four (Supplementary Table S2) as candidate proteins that, potentially, could mediate the intercellular movement of CMV MP through PD. These included immunoglobulin-binding protein (BiP), an ER-located HSP70 (Fig. 1A). Arabidopsis HSP70s are categorized into four classes based on their subcellular localization, and three *BiP* genes are encoded in Arabidopsis (Maruyama *et al.*, 2010) (Fig. 1B). To gain insight into the subcellular localization of the AtBiPs, within PD, we generated constructs of mCherry-fused AtBiP1, 2, and 3. As the possibility was earlier reported concerning potential SP cleavage in BiP (Denecke *et al*., 1991; Marshall *et al.*, 2011), for each construct mCherry was fused between the SP and the rest of that AtBiP coding sequence. As expected, AtBiP1–GFP and AtBiP3–GFP exhibited typical ER network patterns (Supplementary Fig. S1). Interestingly, mCherry–AtBiP1 and –AtBiP2, but not mCherry–AtBiP3, showed punctate fluorescent foci along the cell wall region and were co-localized with CMV MP–GFP, which has been well established to accumulate within PD during the infection process (Ding *et al*., 1995) (Fig. 1C), supporting the hypothesis that AtBiP1 and AtBiP2 are present in PD. TEM-based immunogold labeling assays were conducted on wild-type and *GFP-AtBiP3* transgenic plants and revealed that, as expected, all AtBiP1/2 and GFP–AtBiP3 were located in the ER and that AtBiP1/2, but not GFP–AtBiP3, was detected within PD (Fig. 1D, E).

### AtBiP1/2 signal peptide is necessary for their PD targeting

**Fig. 2.**
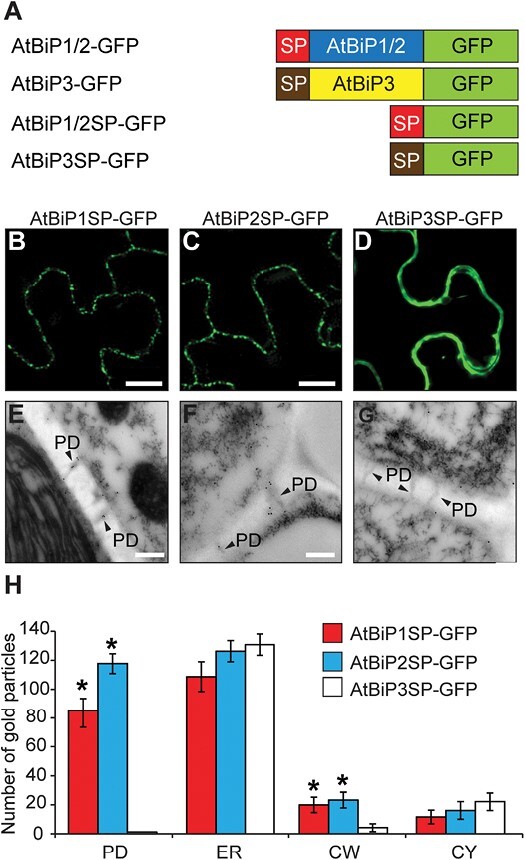
AtBiP1/2 signal peptide is essential for PD targeting. (A) Schematic illustration of chimeric AtBiP constructs used for testing signal peptide (SP) function in PD targeting. Red and brown boxes indicate N-terminal SPs of AtBiP1/2 and AtBiP3, respectively. GFP was fused alone with AtBiP1, 2, or 3 SP (AtBiP1SP–GFP, AtBiP2SP–GFP, or AtBiP3SP–GFP). (B) AtBiP1SP–GFP and (C) AtBiP2SP–GFP signals, not (D) AtBiP3–GFP, were accumulated in puncta at the cell periphery. *AtBiP1SP-GFP*, *AtBiP2SP-GFP*, and *AtBiP3-GFP* constructs were transiently expressed in *N. benthamiana* leaf and observed as a single scan plane, using confocal laser scanning microscopy 3 d after agroinfiltration. Scale bars=10 µm. (E) AtBiP1SP–GFP and (F) AtBiP2SP–GFP, not (G) AtBiP3–GFP, located within PD. Arabidopsis root tissues in which AtBiP1SP–GFP, AtBiP2SP–GFP, or AtBiP3–GFP was expressed were used for TEM-based immunogold labeling with anti-GFP antibody. Arrowheads indicate gold particles in PD. Scale bar=200 nm. (H) Quantification of AtBiP1SP–GFP, AtBiP2SP–GFP, and AtBiP3SP–GFP immunogold signals in all tested sections. More than 50 individual sections collected from root samples were examined to establish the pattern of immunogold labeling. An asterisk above the error bar indicates significant differences in the number of gold particles detected for AtBiP1/2 SP–GFP and AtBiP3SP–GFP, within PD and cell wall, *P*<0.002 based on Student’s *t*-test. PD, plasmodesmata; ER, endoplasmic reticulum; CW, cell wall; CY, cytoplasm.

The BiP proteins possess N-terminal ER SPs, which are required for their translocation into the ER lumen (Tsai *et al.*, 2015). The AtBiP1 and AtBiP2 SPs are highly conserved relative to the AtBiP3 SP (Supplementary Fig. S2A). As AtBiP3 was not detected in PD (Fig. 1C, D), we hypothesize that the AtBiP1 and AtBiP2 SPs contain a motif specific for their ER targeting to PD. To test this hypothesis, we generated mutants in which the AtBiP1/2 SP was either removed (AtBiP1/2ΔSP–GFP) (Supplementary Fig. S2B) or fused to GFP (AtBiP1/2SP–GFP) (Fig. 2A). Consistent with a previous report, deletion of the SPs from AtBiP1/2 yielded a change in their subcellular localization from the ER to the cytoplasm (Supplementary Fig. S2C, D) (Ni *et al.*, 2009). However, the AtBiP1SP–GFP and AtBiP2SP–GFP signals, but not that for AtBiP3SP–GFP, accumulated as puncta at the cell periphery (Fig. 2B–D). Subcellular localization studies, based on immunogold labeling assay using a GFP Ab, revealed that both AtBiP1SP–GFP and AtBiP2SP–GFP were targeted to the PD (Fig. 2E–G); absence of AtBiP3SP–GFP from PD was confirmed by quantitation of immunogold signals (Fig. 2H). Taken together, these findings support the hypothesis that AtBiP1 and AtBiP2 SPs are necessary and contribute to targeting these proteins to the ER located within PD.

### AtERdj2A mediates the association between AtBiP1/2 and CMV MP at the PD ER membrane

**Fig. 3.**
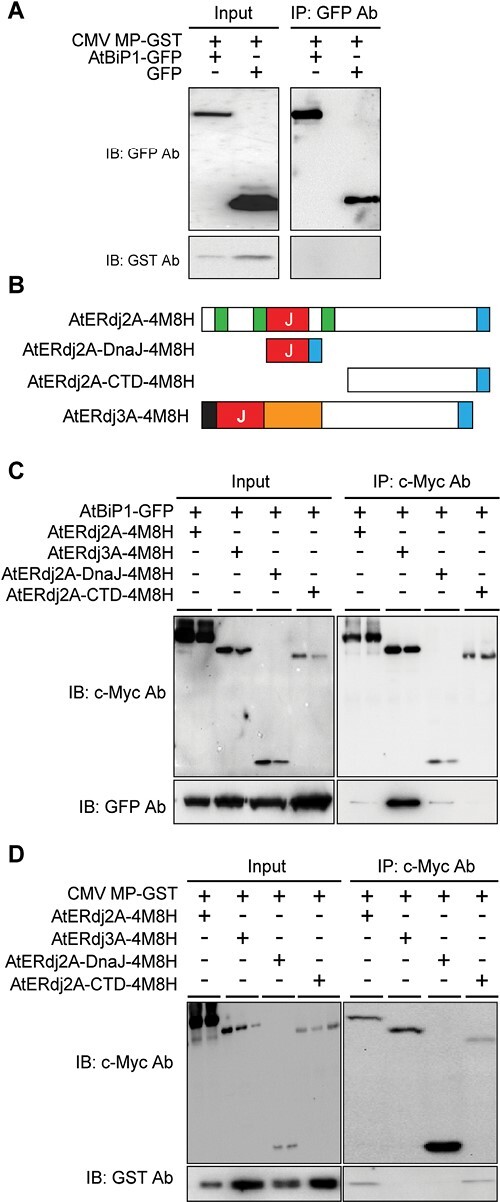
AtERdj2A acts as a contact between AtBiP1 and CMV MP. (A) AtBiP1–GFP did not directly bind to CMV MP–GST. *In planta* recombinant CMV MP–GST, AtBiP1–GFP, or GFP was transiently expressed in *N. benthamiana* leaves and purified with a GST or GFP affinity column. AtBiP1–GFP or GFP was incubated with CMV MP–GST and followed by immunoprecipitation with anti-GFP Ab. The immunoblotting analysis was performed with anti-GFP and GST Ab before (Input) and after immunoprecipitation. (B) Schematic illustration of AtERdj2A and AtERdj3A constructs used to examine the interaction with AtBiP1 and CMV MP. Green, red, black, and orange boxes indicate membrane-spanning regions, the DnaJ domain, the signal peptide, and the G/F-rich domain, respectively. AtERdj2A (AtERdj2A–4M8H), DnaJ domain (AtERdj2A–DnaJ–4M8H), or C-terminal domain (AtERdj2A–CTD–4M8H) was fused with a 4-Myc-8-His (4M8H) tag (blue box) at the C-terminus. (C) The DnaJ domain was involved in the direct interaction between AtBiP1 and AtERdj2A. (D) The CTD domain was involved in the direct interaction between CMV MP and AtERdj2A. (C and D) AtERdj2A–4M8H, AtERdj2A–DnaJ–4M8H, AtERdj2A–CTD–4M8H, or AtERdj3A was transiently expressed with AtBiP1–GFP (C) or CMV MP–GST (D) in *N. benthamiana* leaf and co-immunoprecipitated with anti-c-Myc Ab. Immunoblotting (IB) analyses were performed on extracted total proteins (Input) with anti-c-Myc. Immunoprecipitated (IP) products were examined using immunoblotting with GFP (C) or GST (D) Ab. At least three replicates were used in the co-IP assays, and one representative from the co-IP data is shown in (C) and (D).

As the BiPs are ER luminal proteins, and viral MPs traffic through the cytoplasmic nanochannels of PD (Lucas, 2006; Lucas *et al.*, 2009; Sager and Lee, 2014), it seemed highly unlikely that *in planta*, the CMV MP could interact directly with AtBiP1/2. To test this notion, we transiently expressed a GST-fused CMV MP, AtBiP1/2–GFP, or GFP in *N. benthamiana* leaves, followed by individual protein purification with a GST or GFP affinity column. Next, AtBiP1–GFP or GFP was incubated with CMV MP–GST, and immunoprecipitation was then performed with anti-GFP Ab. Immunoblotting analysis with anti-GFP and anti-GST Abs indicated that the CMV MP–GST did not interact directly with the AtBiP1/2–GFP (Fig. 3A).

This result suggested the presence of an additional protein located in the PD ER which could physically associate between the CMV MP and AtBiP1/2. Two other ER-located proteins, AtERdj2A and AtERdj2B (Supplementary Fig. S3), were identified in our CMV MP PECP co-IP assays. Here, the low AtERdj2A/B sequence coverage of 2–2.8% (Supplementary Table S2) probably reflects their being integral membrane proteins. AtERdj2A/B are Sec63p orthologs in Arabidopsis, and this integral ER membrane protein contains three membrane-spanning segments and a DnaJ domain (Fig. 3B) (Yamamoto *et al.*, 2008). The DnaJ and CTDs of the yeast Sec63p face the ER lumen and cytoplasm, respectively (Rothblatt *et al.*, 1989; Sadler *et al.*, 1989; Voith von Voithenberg *et al.*, 2021). A bioinformatic analysis (https://services.healthtech.dtu.dk/service.php?TMHMM2.0) predicted that the AtERdj2A/B DnaJ domain and CTD also locate towards the ER lumen and cytoplasm, respectively. Furthermore, the yeast Sec63p DnaJ domain was previously shown to interact with the yeast BiP (Tyedmers *et al.*, 2000).

Based on these findings, we hypothesized that AtERdj2A/B function in the association between AtBiP1/2 and the CMV MP at the PD ER membrane and, to this end, the AtERdj2A/B DnaJ domain interacts with AtBiP1/2, located in the ER lumen, and the CTD interacts with the CMV MP, in the cytoplasmic channel of the PD. Given that AtERdj2A and AtERdj2B are highly conserved (Supplementary Fig. S4), and that both proteins were detected in our CMV MP-based PECP co-IP assays. We tested this hypothesis by performing *in planta* immunoprecipitation assays using AtERdj2A. Here, Myc-8His (4M8H)-tagged AtERdj2A (AtERdj2A–4M8H) or truncated forms of AtERdj2A (DnaJ, AtERdj2A–DnaJ–4M8H; CTD, AtERdj2A–CTD–4M8H) (Fig. 3B) were transiently expressed along with AtBiP1–GFP or CMV MP–GST in *N. benthamiana* leaves, followed by co-IP with an anti-c-Myc Ab (Fig. 3C, D). Another ER-located DnaJ protein, AtERdj3A, which is also present in the ER lumen and interacts with AtBiP1, was used as a positive control (Yamamoto *et al.*, 2008; Ma *et al.*, 2015).

Based on these assays, AtERdj2A–4M8H was immunoprecipitated with AtBiP1–GFP (Fig. 3C). In addition, the AtERdj2A DnaJ domain, but not its CTD, interacted with AtBiP1–GFP (Fig. 3C) and, importantly, CMV MP–GST bound to the AtERdj2A CTD, but not to its DnaJ domain (Fig. 3D). The GFP control was not involved in any interaction with AtERdj2A (Supplementary Fig. S5), and nor was there any interaction between AtERdj3A–4M8H and either AtBiP1–GFP or CMV MP–GST (Fig. 3C, D). These results offered support for our model in which AtERdj2A interacts with both AtBiP1 and CMV MP to act as a mediator for AtBiP1–CMV MP association at the PD ER membrane.

### AtBiP1/2 and AtERdj2 functioned in CMV infection

**Fig. 4.**
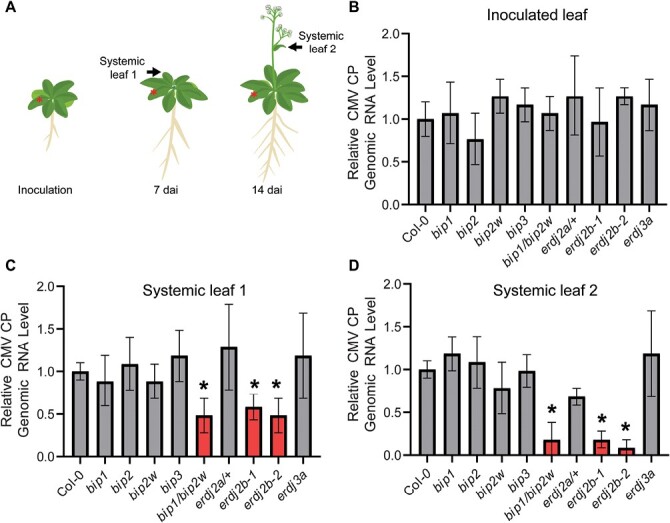
AtERdj2B knockout mutants display decreased CMV infectivity. (A) Schematic diagram of Arabidopsis plants after CMV inoculation. Fourteen-day-old Arabidopsis plants were inoculated with CMV-Fny using an agroinfiltration method. Arabidopsis leaves were collected 7 d and 14 d after CMV-Fny and mock inoculation. Asterisks indicate virus-inoculated leaves. Black arrows indicate systemic rosette (systemic leaf 1) and cauline leaves (systemic leaf 2). (B–D) Quantification of CMV CP RNA accumulation in wild-type and knockout mutants of AtBiPs, AtERdj2, and AtERdj3A using qRT-PCR. Total RNA was extracted at 7 d after CMV inoculation (dai) from inoculated (B) and systemic rosette (C) leaves and 14 dai from systemic cauline leaves (D) of Mock- or CMV-inoculated Arabidopsis plants. The mean value of CMV CP RNA in the wild type was used as the reference (1.0). Error bars represent the SD (*n*=6). Asterisks above the error bars indicate statistical significance between the *CMV CP* level and the wild type at *P*<0.05 (Student’s *t*-test).

To further assess the role of AtBiP1/2 and AtERdj2A/B in CMV infection, we next examined whether mutations in AtBiP1/2 or AtERdj2A/B would negatively impact CMV infectivity compared with wild-type Arabidopsis (Fig. 4). For analyses of AtBiP function in CMV spread, we used the null alleles of *bip1-4* (*bip1*), *bip2-1* (*bip2*), and *bip3* (*bip3-1*). As the *bip1/bip2* double mutant is lethal (Maruyama *et al.*, 2010), we crossed *bip1* with *bip2-2* (*bip2w*), which is a knockdown allele, to generate a *bip1/bip2w* double mutant line with a knockdown level of *AtBiP2* expression. Consistent with the previous report, all tested AtBiP homozygous mutant lines did not exhibit any significant phenotypic differences compared with the wild type in terms of plant growth (Maruyama *et al.*, 2010); similarly, no obvious phenotypic differences were observed between the wild type and the *bip1/bip2w* double mutant line.

Fourteen-day-old Arabidopsis plant lines were infected with CMV-Fny using the agroinfiltration method (Zhang *et al.*, 2017). Inoculated and systemic rosette leaves were harvested at 7 dai and systemic cauline leaves at 14 dai (Fig. 4A). The qRT-PCR assays were performed to evaluate CMV RNA accumulation in these infected plant lines. A similar level of CMV CP RNA was detected in the wild type and *bip1*, *bip2*, *bip2w*, and *bip3* mutants, in both the inoculated and systemic leaves (Fig. 4B–D). However, in systemic leaves of the *bip1/bip2w* double mutant, CMV CP RNA accumulation was reduced significantly compared with the wild type (Fig. 4C, D).

Next, we assessed whether the AtBiP1/2–AtERdj2A and CMV MP–AtERdj2A interactions play a role in CMV viral infectivity. Here, we note that AtERdj3A was not identified in our CMV MP PECP co-IP assays (Supplementary Table S2), although it could interact with AtBiP1/2 in the ER lumen (Fig. 3C) (Ma *et al.*, 2015). Consistent with these findings, the AtERdj3A mutant, *erdj3a*, showed similar CMV infectivity to the wild type in both inoculated and systemic leaves, suggesting that AtERdj3A is not involved in CMV viral spread (Fig. 4B–D) (Ma *et al.*, 2015). The homozygous *erdj2a-1* mutant is lethal (Yamamoto *et al.*, 2008); therefore we tested whether a heterozygous AtERdj2A mutant (*erdj2a/+*) displayed evidence of inhibition in CMV infectivity. However, *erdj2a/+* plants showed no significant reduction in CMV CP RNA levels in inoculated and systemic leaves (Fig. 4B–D).

In Arabidopsis, AtERdj2B is also present in the ER membrane and, as mentioned above, has high amino acid sequence similarity (85.3%) to AtERdj2A (Supplementary Fig. S4). As AtERdj2B was also detected as one of the CMV MP-interacting proteins, we next tested CMV infectivity in the *erdj2b-1* and *erdj2b-2* null mutant lines (Yamamoto *et al.*, 2008). These plants were inoculated with CMV-Fny and, interestingly, compared with the wild type, these mutants all had reduced CMV CP RNA levels in their systemic, but not in their inoculated, leaves (Fig. 4B–D).

The CMV strains are categorized as subgroup I and II, based on sequence homology at the nucleotide level, and these two CMV subgroups display different symptom severity (Mochizuki and Ohki, 2012). To test whether the functions of AtBiP1/2 and AtERdj2A/B are conserved in viral spread of both CMV subgroup I and II, the above-described Arabidopsis mutants were challenged with the CMV-Q strain, which belongs to CMV subgroup II, followed by qRT-PCR to detect the level of CMV CP RNA (Supplementary Fig. S6). As with viral infectivity with CMV-Fny from subgroup I, in these mutants (Fig. 4) lower levels of CMV CP RNA were also detected in *bip1/bip2w*, *erdj2b-1*, and *erdj2b-2* compared with the wild type (Supplementary Fig. S6), suggesting that AtBiP1/2 and AtERdj2A/B mediate viral spread of both CMV subgroups I and II.

**Fig. 5.**
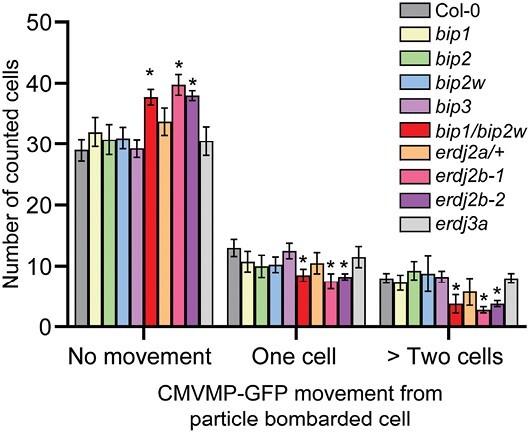
Mutation of AtBiP1/2 or AtERdj2b compromised intercellular movement of CMV MP–GFP. Cells with a CMV MP–GFP fluorescent signal were quantified in Arabidopsis wild-type and mutant lines 40 h after particle bombardment. No movement, CMV MP–GFP signal was detected in only bombarded cells; one cell, CMV MP–GFP signal was detected in the cell adjoining the bombarded cell; >two cells, CMV MP–GFP signal was detected in two or more cells away from the bombarded cell. Six replicates were used in these CMV MP–GFP trafficking assays and a total of 50 cells were examined in each replicate. Values are reported as means ±SD (*n*=6). Asterisks above the error bars indicate significant differences in counted cell numbers with respect to the wild type at *P*<0.05 (Student’s *t*-test).

AtBiP1/2 and AtERdj2A/B were identified as CMV MP-interacting proteins within the PD (Supplementary Table S2). As these proteins appear to mediate the control over CMV spread in Arabidopsis, we hypothesized that AtBiP1/2 and AtERdj2A/B regulate intercellular movement of CMV MP through PD. To test this notion, CMV MP–GFP was transiently expressed in Arabidopsis leaves using biolistic particle bombardment (Ueki *et al.*, 2009; Taoka *et al*., 2007) (Fig. 5; Supplementary Fig. S7). Compared with the wild type, higher numbers of fluorescent cells, in which CMV MP–GFP was only detected in bombarded cells, were quantified in *bip1/bip2w*, *erdj2b-1*, and *erdj2b-2* mutant lines, and the mutations in AtBiP1/2 and AtERdj2A/B appeared to compromise cell-to-cell movement of CMV MP–GFP into the neighboring cells (Fig. 5). Taken together with our viral infectivity assays, these findings offered support for the hypothesis that AtBiP1/2 and AtERdj2A/B are necessary for cell-to-cell trafficking of CMV MP.

## Discussion

### AtBiP1/2 are targeted to the ER within PD

In this study, co-IP assays allowed us to identify CMV MP-interacting proteins from an Arabidopsis PECP (Fig. 1A; Supplementary Table S2). Importantly, the seven identified proteins were all listed in previously reported PD proteomes (Fernandez-Calvino *et al.*, 2011; Kraner *et al.*, 2017). The three *BiP* genes, *AtBiP1*, *AtBiP2*, and *AtBiP3*, have been identified in Arabidopsis (Fig. 1B), and AtBiP1 and AtBiP2 share 99% identity at the amino acid sequence level (Noh *et al.*, 2003). This high sequence conservation challenged our ability to map the identified peptides to either AtBiP1 or AtBiP2 (Supplementary Table S2). Hence, we hypothesized that AtBiP1 or AtBiP2, or both, are located at the ER within the PD to mediate an interaction with CMV MP.

Co-localization assays and TEM-based immunogold labeling imaging demonstrated that mCherry-tagged AtBiP1 and AtBiP2, but not AtBiP3, accumulated in puncta at the cell wall and were detected in PD (Fig. 1D, E). The *AtBiP1* and *AtBiP2* genes have been shown to be constitutively expressed, and their proteins have been found to be located within the ER lumen; however, AtBiP3 is detected only during ER stress conditions (Noh *et al.*, 2003). In this regard, it is interesting to note that, in our *AtBiP3*-*GFP* overexpression lines, it was not detected in PD, suggesting that a protein sequence-specific mechanism may operate to target AtBiP1 and AtBiP2 to the PD ER. As AtBiP1 and AtBiP2 have been shown to be functionally redundant in Arabidopsis (Maruyama *et al.*, 2010) and, given that in the individual *bip1* and *bip2* mutant lines CMV infection was unaffected, these findings offer further support for the hypothesis that both AtBiP1 and AtBiP2 are present within the PD ER (Fig. 1D, E).

The BiP SPs function in ER targeting (Park *et al.*, 2019) and, given the AtBiP1 and AtBiP2 high sequence homology (99%), as expected, only two amino acids were different in their SPs; however, the AtBiP3 SP had only 18% identity with AtBiP1 and AtBiP2 SPs (Supplementary Fig. S2). The high AtBiP3 SP sequence divergence from the AtBiP1 and AtBiP2 SPs may underly their function in the targeting of AtBiP1/2 to the ER within PD. Support for this notion is provided by the finding that both AtBiP1/2 deletion mutants failed to accumulate at the cell periphery (Supplementary Fig. S2), and that both SPs of AtBiP1 and AtBiP2, but not that of AtBiP3 SP, were necessary and sufficient for the localization of AtBiP1SP- and AtBiP2SP-tagged GFP to the PD (Fig. 2B–H). Of note, the AtBiP1 and AtBiP2 SPs contained a different sequence composition from those present on the Plasmodesmata Germin-like Proteins (PDGLPs) and the Plasmodesmata-located Proteins (PDLPs), both of which were previously reported to contain PD-targeting motifs (Thomas *et al.*, 2008; Ham *et al.*, 2012). This suggests an independent pathway for the evolution of PD-targeting SPs. The mechanism involved in PD targeting via AtBiP1SP and AtBiP2SP remains to be further elucidated, but these signal sequences might be involved in an interaction with other ER-located proteins, such as AtERdj2A and AtERdj2B, and play a role in sequestration of AtBiP1/2 to the PD.

### Association of AtBiP1/2–AtERdj2A–CMV MP proteins at PD

Even though AtBiP1 and/or AtBiP2 were co-immunoprecipitated with CMV MP (Supplementary Table S2; Fig. 1A), the CMV MP did not directly interact with AtBiP1 (Fig. 3A). The co-IP assay can identify both direct and indirect interacting proteins within protein complexes (Miernyk and Thelen, 2008). As BiPs are ER-luminal HSP70s, we hypothesized that other CMV MP co-IP products were involved in the association at the PD ER membrane between the ER luminal AtBiP1 and cytoplasmic-located CMV MP. AtERdj2A and AtERdj2B were identified as CMV MP co-IP products (Supplementary Table S2): AtERdj2A/B are yeast Sec63p orthologs and are ER integral membrane proteins, with a DnaJ domain that functions with BiP to mediate protein translocation (Rothblatt *et al.*, 1989; Sadler *et al.*, 1989; Yamamoto *et al.*, 2008; Voith von Voithenberg *et al*., 2021). Thus, we hypothesized that AtERdj2A/B locates in the PD ER membrane to associate between AtBiP1/2 and CMV MP. Consistent with this model, AtERdj2A–GFP co-localized with both CMV MP–mCherry and AtBiP1–mCherry (Supplementary Fig. S3).

*In vivo* pull-down assays revealed that AtBiP1 and CMV MP interact with the DnaJ and CTD domains of AtERdj2A, respectively (Fig. 3C, D). Several studies demonstrated that ERdj2 associates with BiP as a co-chaperone: here, the DnaJ and CTD domains of the yeast ERdj2 interact with BiP and Sec62, respectively, and play a role in protein transport across the ER membrane (Tyedmers *et al.*, 2000; Muller *et al.*, 2010; Gao *et al.*, 2012; Hassdenteufel *et al.*, 2018; Griffith and Holmes, 2019). Our results are consistent with this model in terms of an AtERdj2A–AtBiP1/2 interaction at the PD ER membrane; however, CMV MP interacts with the AtERdj2A CTD, not to permit its translocation into the ER lumen but rather to mediate its trafficking along the PD ER for cell-to-cell movement.

### ER-mediated pathway for cell-to-cell trafficking of viral movement proteins through PD

The CMV infection assays, using agroinfiltration methods to inoculate CMV into Arabidopsis, revealed that AtBiP1/2 and AtERdj2 mutants restricted both CMV subgroup I and II spread (Fig. 4; Supplementary Fig. S6). In the inoculated leaves, all mutants used in this study had CMV RNA levels equivalent to that of the wild type. As agrobacteria carrying the CMV constructs were infiltrated into the entire area of inoculated leaves, excluding the vascular tissue, CMV could be expressed within these inoculated tissues. Thus, AtBiP1/2 and AtERdj2A/B appeared not to be involved in CMV amplification at the level of mesophyll-infected cells. Furthermore, as the AtBiPs homozygous *bip1/bip2w*, *erdj2a/+*, and *erdj2b* mutant lines lacked any observable phenotypic difference(s) from wild-type plants, these mutations may not affect PD function regarding intercellular trafficking of non-cell-autonomous factors involved in plant growth and development. It is also a challenge to evaluate PD structure in *bip1/bip2* and *erdj2a* due to lethality of these mutant lines (Yamamoto *et al.*, 2008; Maruyama *et al.*, 2010). However, *bip1/bip2w* and *erdj2b* mutants displayed retarded CMV infection in systemic leaves (Fig. 4; Supplementary Fig. S6). Furthermore, AtBiP1/2 and AtERdj2A/2B appear to regulate intercellular trafficking of CMV MP, which was compromised in those mutant lines (Fig. 5). These findings suggest that, in these mutant lines, inhibition of PD-mediated CMV viral RNA trafficking, from bundle sheath cells into the phloem translocation stream, may have resulted in the observed delay in a systemic viral spread.

Based on their subcellular localization in plant cells, Hsp70s were categorized into four groups (Fig. 1B) that function as chaperones to stabilize protein folding and translocation across organellar membranes (Karlin and Brocchieri, 1998; Rosenzweig *et al.*, 2019; Berka *et al.*, 2022). Several studies have proposed Hsp70 roles in viral replication and intercellular trafficking. For example, Chinese wheat mosaic furovirus, cowpea severe mosaic virus, tomato bushy stunt virus, and potato virus Y utilize Hsp70 for their viral replication in host plants (Pogany *et al.*, 2008; Paiva *et al*, 2016; Varela *et al.*, 2017; Yang *et al.*, 2017). In addition, Hsp70s form protein complexes with viral MPs and coat proteins to mediate cell-to-cell transport through PD (Aoki *et al.*, 2002; Nelson and Citovsky, 2005; Krenz *et al.*, 2010). In this regard, although AtBiP1 associates with CMV MP through AtERdj2A/B, it is possible that, as an ER-luminal Hsp70, it could confer stability to the binding of AtERdj2A/B to the CMV MP during cell-to-cell transport of bound viral RNA through the PD.

The *aterdj2a-1* heterozygous mutant (*erdj2a/+*) was used to examine the dosage effect of an essential gene, *AtERdj2A*, in CMV infection (Fig. 4; Supplementary Fig. S6). Here, no significant difference in CMV infectivity was observed, relative to the wild type, in inoculated and systemic leaves of *erdj2a/+*, consistent with the presence of a functional paralog of AtERdj2A in Arabidopsis. As the mutation of AtERdj2A, but not AtERdj2B, resulted in lethality in an *erdj2a* homozygous mutant, and the expression level of *AtERdj2A* was higher than that of *AtERdj2B* (Winter *et al.*, 2007; Yamamoto *et al.*, 2008), it has been proposed that AtERdj2A is essential in plant growth, but that AtERdj2B functions in supporting roles for AtERdj2A in the ER. In addition, AtERdj2A and AtERdj2B have high sequence similarity (85.3%) in amino acid residues (Supplementary Fig. S4) and both are integral ER membrane proteins (Yamamoto *et al.*, 2008); therefore, it is plausible that AtERdj2A and AtERdj2B share functionality in the ER. Interestingly, lower levels of CMV CP RNA were detected in the *erdj2b* mutants, compared with the wild type (Fig. 4; Supplementary Fig. S6), even though *AtERdj2A* is normally expressed in the *erdj2b* mutant lines (Yamamoto *et al.*, 2008). Thus, it may be that AtERdj2B, rather than AtERdj2A, functions as the major component in cell-to-cell trafficking of CMV MP, and presumably for PD-mediated trafficking of other non-cell-autonomous complexes in plants.

### Model of ER-mediated CMV MP trafficking pathway through PD

**Fig. 6.**
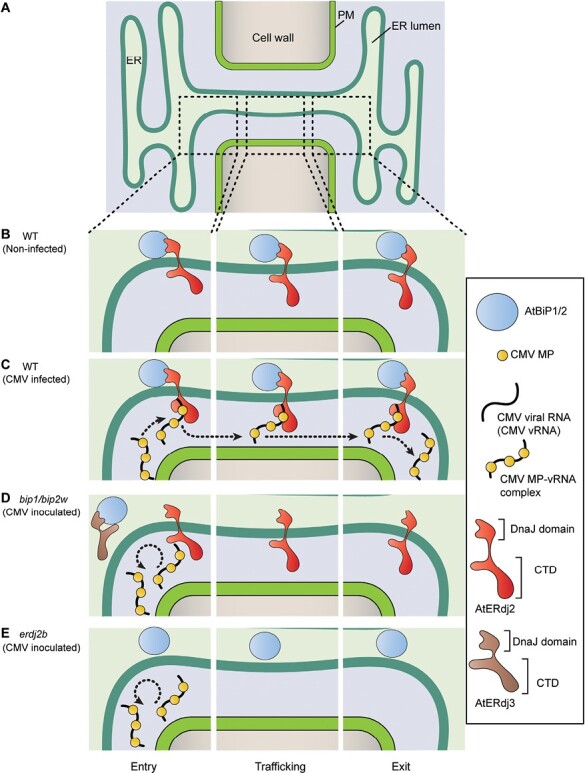
Schematic model illustrating the role of AtBiP1/2 and AtERdj2A/2B in CMV MP trafficking through PD. (A) Schematic longitudinal PD structure illustrating the continuity of appressed ER across the cell wall. Dotted squares indicate the regions of the appressed ER within PD to illustrate the models proposed in (B), (C), (D), and (E). (B) In control wild-type plants (no CMV infection), AtBiP1/2 and AtERdj2 interact with each other and are located in the PD ER lumen and ER membrane, respectively. (C) AtERdj2 establishes a connection between AtBiP1/2 and CMV MP. AtBiP1/2 located in the PD ER lumen interact with the ER membrane-anchored AtERdj2. CMV MP binds to this AtBiP1/2–AtERdj2 complex for its traffic into the neighboring cells. (D) In the *bip1/bip2w* mutant, AtERdj3A interacts with AtBiP2 in the ER lumen, thereby restricting efficient PD targeting of AtBiP2 to the PD ER lumen. (E) In the *erdj2b* mutant, AtERdj2B is absent from the PD ER membrane, thereby limiting the opportunity for CMV MP to bind to AtERdj2, which results in an inefficient cell-to-cell transport of CMV MP (and its viral RNA).

Several models have been proposed with regard to ER-mediated cell-to-cell movement of viral MPs through PD. TMV MP, associated with viral RNA, accumulates in ER-derived vesicles that retain β-1,3-glucanase to degrade callose at PD, for its trafficking through PD (Reichel and Beachy, 1998; Epel, 2009; Guenoune-Gelbart *et al*., 2008; Zavaliev *et al.*, 2013). Synaptotagmin (SYT) A/1 is one of the ER–plasma membrane contact site proteins and has been proposed to form protein complexes with TMV or turnip vein clearing virus (TVCV) MPs for their PD targeting and intercellular movement (Uchiyama *et al.*, 2014; Levy *et al.*, 2015). As these models have not been proposed for cell-to-cell movement of MPs in the Bromoviridae family to which CMV belongs and as PD appear to establish selective transport pathways for viral MPs (Lee *et al.*, 2003), here, based on our findings, we propose a new model in which an integral AtBiP1/2–AtERdj2A/B complex binds to CMV MP, thereby establishing an AtBiP1/2–AtERdj2A/B–CMV MP association to mediate the intercellular trafficking of a CMV MP–viral RNA complex through PD (Fig. 6A–C). A recent study reported that the *N. benthamiana* BiP4 can associate with triple-gene-block protein 3 (TGBp3), which functions as the potyvirus MP, and serves in intracellular movement of bamboo mosaic virus for PD targeting (Huang *et al.*, 2023). Therefore, we cannot exclude the possibility that AtBiP1/2–AtERdj2A/B may also play a role in the PD targeting of CMV, via the ER network. In the *bip1/bip2w* mutant plants, the *AtBiP2* transcript level is lower than in the wild type and, as AtERdj3A can interact with AtBiP2 in the ER lumen within the cytoplasm (Maruyama *et al.*, 2010; Ma *et al.*, 2015), AtERdj3A could compromise PD targeting of AtBiP2 in the *bip1/bip2w* mutant (Fig. 6D). Thus, in *erdj2b* mutant plants, stability of the AtBiP1/2–AtERdj2A/B–CMV MP–viral RNA complexes would be compromised (Fig. 6E). However, as these mutants did not fully block CMV spread (Fig. 4; Supplementary Fig. S6), we cannot exclude the possibility that other ER factors might also be involved in the association with CMV MP–AtERdj2A/B during the intercellular movement of CMV MP through PD.

In this study, we provide insight into the role of the PD ER system in mediating intercellular trafficking of a viral MP. Future studies will be directed at determining the role of this BiP1/2–ERdjA/B pathway in cell-to-cell movement of endogenous non-cell-autonomous signaling agents.

## Supplementary data

The following supplementary data are available at *JXB* online.

Fig. S1. AtBiP1 and AtBiP3 locate at the ER.

Fig. S2. The signal peptides (SPs) of AtBiP1 and AtBiP2 play a role in PD targeting.

Fig. S3. AtERdj2A co-localizes with CMV MP and AtBiP1 in the cell wall.

Fig. S4. Sequence alignment between AtERdj2A and AtERdj2B.

Fig. S5. GFP does not interact with AtERdj2A or AtERdj3A.

Fig. S6. Decreased CMV-Q infectivity in AtERdj2B knockout mutant plants.

Fig. S7. Accumulation of CMVMP–GFP signal in particle-bombarded cells in Arabidopsis Col-0 leaves.

Table S1. List of primers used in this study.

Table S2. List of CMV MP-interacting proteins identified in an Arabidopsis PECP assay.

erad190_suppl_Supplementary_Figures_S1-S7_Tables_S1-S2

## Data Availability

The raw datasets used for co-IP analysis that support the findings of this study are available in the Dryad Digital Repository at https://doi.org/10.5061/dryad.3ffbg79pn (Ham *et al.*, 2023).

## Abbreviations

CMV: cucumber mosaic virus
ER: endoplasmic reticulum
MP: movement protein
PD: plasmodesmata
SP: signal peptide
