# Supplementary material for: Plasmodesmal endoplasmic reticulum proteins regulate intercellular trafficking of cucumber mosaic virus in Arabidopsis
Source: J Exp Bot. 2023 May 21;74(15):4401–14. doi: 10.1093/jxb/erad190 (PMC10838158; doi:10.1093/jxb/erad190)
Supplement: erad190_suppl_Supplementary_Figures_S1-S7_Tables_S1-S2 [file erad190_suppl_supplementary_figures_s1-s7_tables_s1-s2.pdf]

Supplementary data

Figure S1

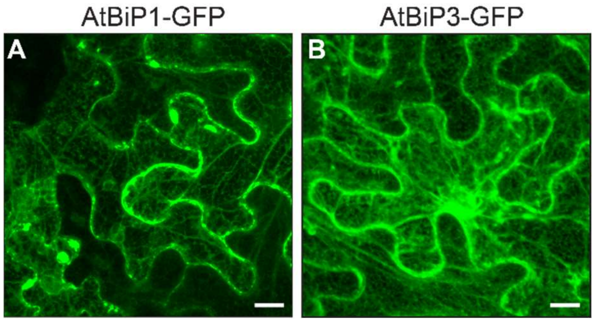

Figure S1. AtBiP1 and AtBiP3 locate at the ER.

AtBiP1-GFP (A) and AtBiP3-GFP (B) were transiently expressed in *N. benthamiana* leaves. Epidermal cells of *N. benthamiana* were imaged using a confocal laser scanning microscope operating in maximal z-projection mode; 20 1 µm optical images were used to form an 18 µm z-stack image. Bars = 10 µm.

Figure S2

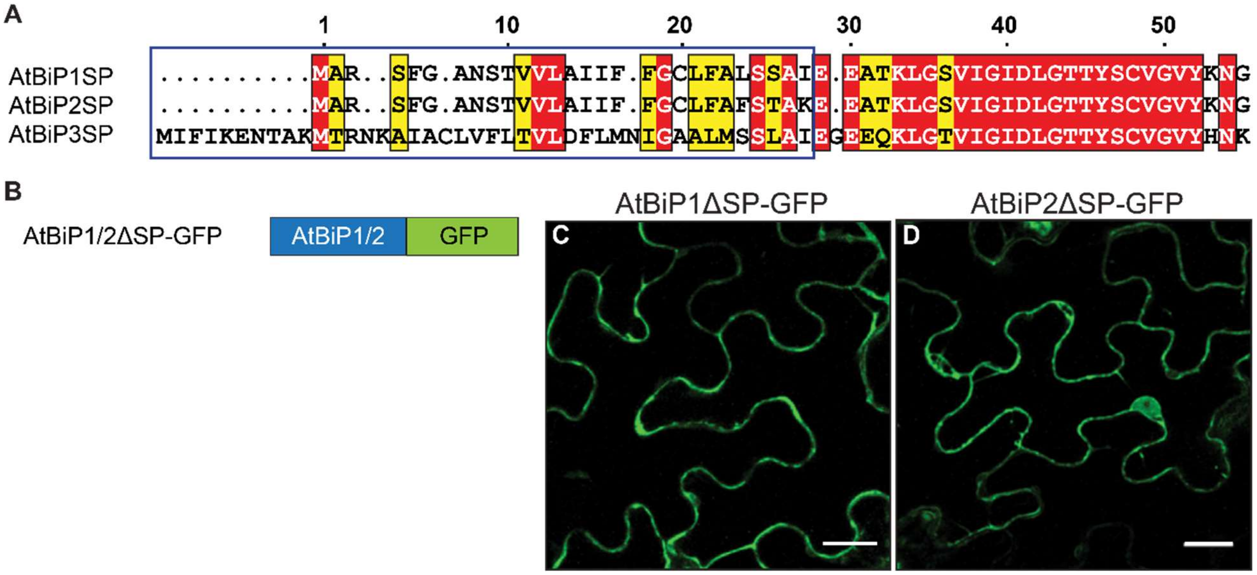

Figure S2. **The AtBiP1 and AtBip2 signal peptides (SPs) play a role in their PD targeting.**

(A) SP sequence alignment of AtBiP1, AtBiP2 and AtBiP3. The blue box indicates predicted SP of AtBiPs. Note that 2 amino acid sequences were different in the SP region between AtBiP1 and AtBiP2. **(B)** Schematic illustration of GFP-fused AtBiP1 or AtBiP2 without SP (AtBiP1/2ΔSP-GFP). **(C)** SP-deleted forms of AtBiP1 and AtBiP2 did not display puncta accumulation pattern at the cell periphery. AtBiP1ΔSP-GFP and **(D)** AtBiP2ΔSP-GFP were agroinfiltrated into *N. benthamiana* and GFP signals were observed as a single scan plane, using a CLSM. Bars = 10 μm.

Figure S3

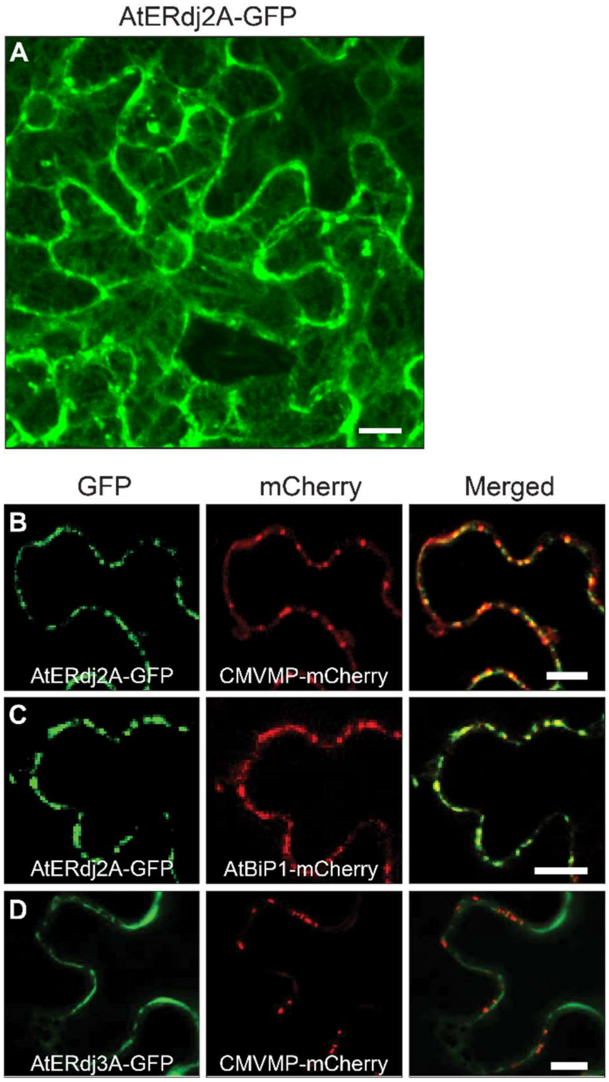

Figure S3. AtERdj2A colocalizes with CMV MP and AtBiP1 in the cell wall

(A) AtERdj2A located at ER. AtERdj2A was transiently expressed in *N. benthamiana* leaves. Epidermal cells of *N. benthamiana* were imaged using a confocal laser scanning microscope operating in maximal z-projection mode; 20 1 μm optical images were used to form an 18 μm z-stack image. Bars = 10 μm. **(B)** AtERdj2A-GFP colocalized with CMV MP-mCherry at the cell periphery. **(C)** AtERdj2A-GFP accumulated with AtBiP1-mCherry in puncta at the cell periphery. The *AtERdj2A-GFP* construct was co-infiltrated with (B) *CMV MP-mCherry*, or (C) *AtBiP1-mCherry* into *N. benthamiana* leaves. Yellowish signals in merged images represent

colocalization of AtERdj2A-GFP with CMV MP-mCherry, or AtBiP1-mCherry. **(D)** AtERdj3A-GFP did not colocalized with CMV MP-mCherry in the cell wall. AtERdj3A-GFP was transiently expressed with CMV MP-mCherry in *N. benthamiana* leaves. Epidermal cells of *N. benthamiana* were imaged, in a single scan plane, using a confocal laser scanning microscope. Bars = 10 μm.

Figure S4

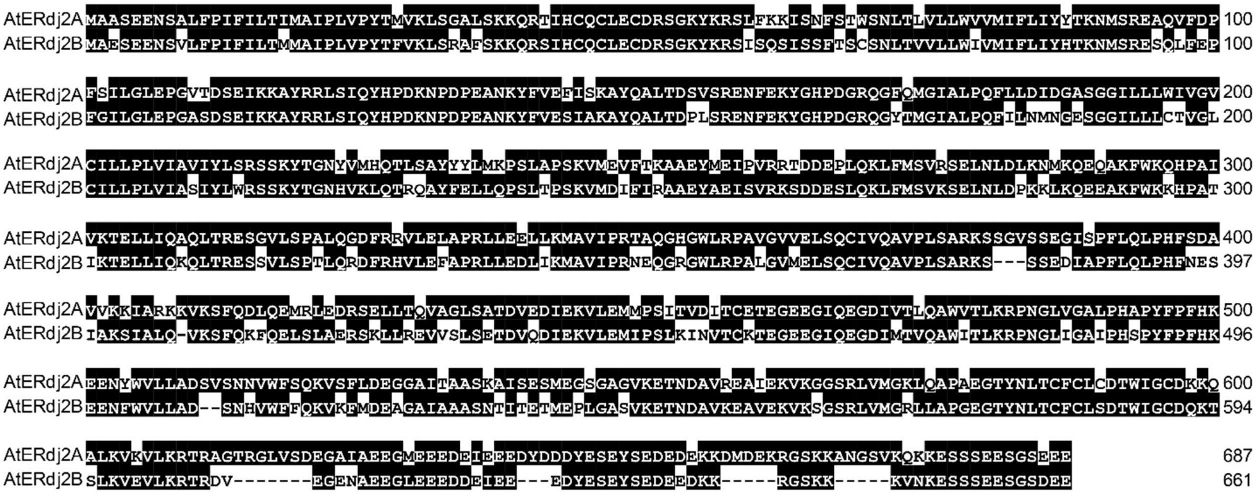

Figure S4. **Sequence alignment between AtERdj2A and AtERdj2B**

Amino acid sequences of AtERdj2A and AtERdj2B were aligned, using the Cluster W method in MEGA X software.

Figure S5

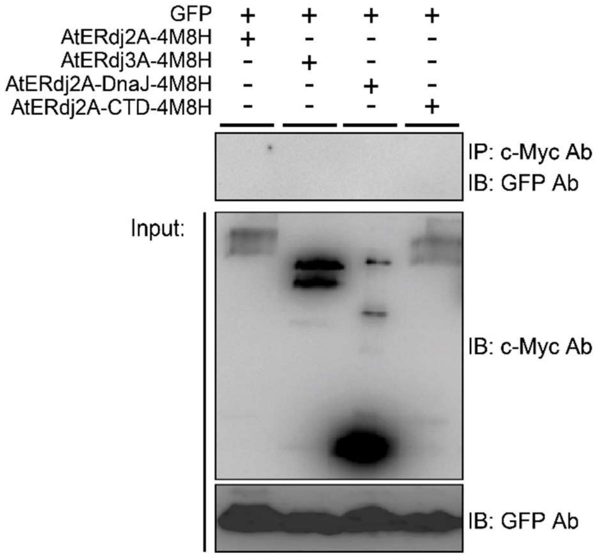

Figure S5. **GFP does not interact with AtERdj2A or AtERdj3A**

GFP was co-agroinfiltrated with AtERdj2A-4M8H, AtERdj2A-DnaJ-4M8H, AtERdj2A-CTD-4M8H, or AtERdj3A in *N. benthamiana* leaves and co-immunoprecipitation was performed with anti-c-Myc Ab. Extracted total proteins (Input) were examined in immunoblotting (IB) assays with anti-c-Myc or anti-GFP Ab. 4M8H-tagged proteins were immunoprecipitated (IP) with anti-c-Myc Ab, followed by immunoblotting analysis with anti-GFP Ab.

Figure S6

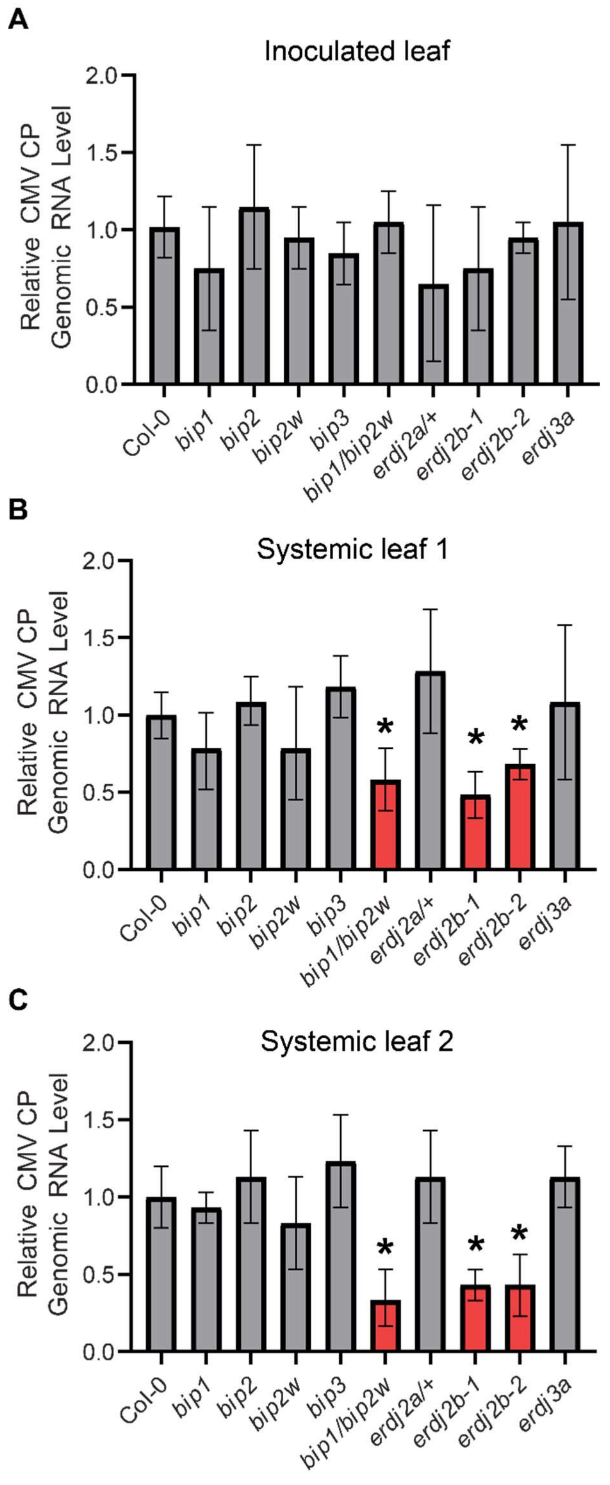

Figure S6. Decreased CMV-Q infectivity in AtERdj2B knockout mutant plants

(A-C) Evaluation of CMV-Q CP RNA accumulation in wild-type and knockout mutants of AtBiPs, AtERdj2 and AtERdj3A, using qRT-PCR. Fourteen-day old Arabidopsis plants were used for CMV-Q inoculation. Total RNA was extracted at 7 dai from inoculated **(A)** and systemic rosette **(B)** leaves and 14 dai from systemic cauline leaves **(C)** of Mock- or CMV-inoculated Arabidopsis plants. The mean value of CMV-Q CP RNA in the wild-type was used as the reference (1.0). Error bars represents the standard deviation (SD; n = 6). Asterisks, above the error bars, indicate statistical significance in *CMV CP* level with wild-type at $p < 0.05$ (Student's t-test).

Figure S7

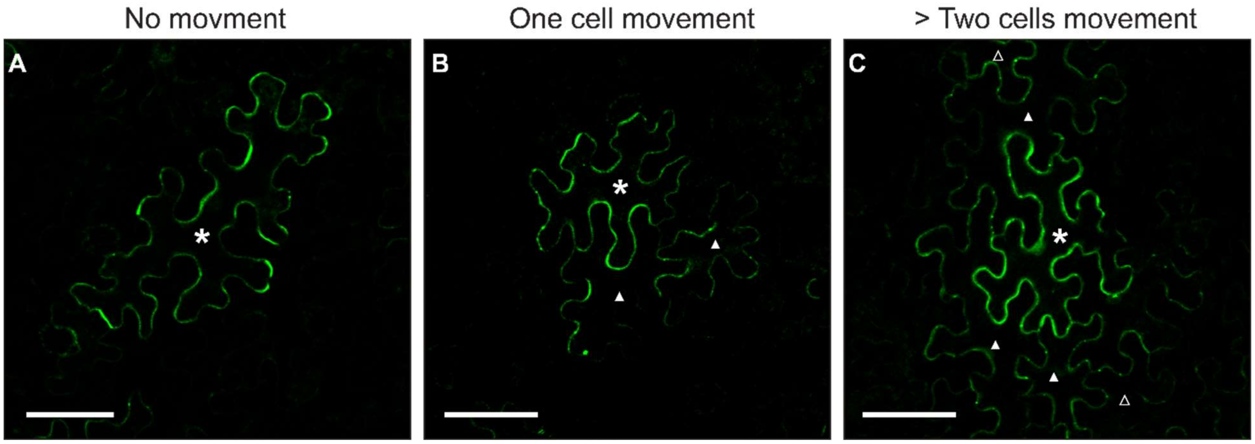

Figure S7. Accumulation of CMVMP-GFP signal in particle-bombarded cells in Arabidopsis Col-0 leaves.

Arabidopsis leaves were bombarded with a CMV MP-GFP construct. Confocal microscopy was used to observe particle-bombarded cells after 40-h incubation period. Asterisks indicate bombarded cells. White closed and open darts indicate accumulation of CMV MP-GFP signal at the neighboring cells and at least two cells away from bombarded cells, respectively. Bars = 50 μm.

Table S1. List of PCR primers used in this study

Primer name	Oligonucleotide sequence
AtBIP1SP-Cherry-R	5'-CCTCGCCCTTGCTCACCATCGCAAATAAACATCCGAAG-3'
Cherry-AtBIP1SP-F	5'-CTTCGGATGTTTATTTGCGATGGTGAGCAAGGGCGAGG-3'
AtBIP1SP-F	5'-ATGGCTCGCTCGTTTGGAGCTAAC-3'
AtBIP1-R	5'-CTAGAGCTCATCGTGAGACTCATC-3'
AtBIP2SP-Cherry-R	5'-CCTCGCCCTTGCTCACCATCGCAAATAAACATCCGAAG-3'
Cherry-AtBIP2-SP-F	5'-CTTCGGATGTTTATTTGCGATGGTGAGCAAGGGCGAGG-3'
AtBIP2SP-F	5'-ATGGCTCGCTCGTTTGGAGCTAAC-3'
AtBIP2-R	5'-CTAGAGCTCATCGTGAGACTCATC-3'
AtBiP3SP-Cherry-R	5'-CCTCGCCCTTGCTCACCATCGTCCCCAGTTTCTGCTCTTC-3'
AtBiP3SP-Cherry-F	5'-GAAGAGCAGAAACTGGGGACGATGGTGAGCAAGGGCGAGG-3'
AtBiP3SP-GFP-R	5'-GTTCTTCTCCTTTGCCCATCGTCCCCAGTTTCTGCTCTTC-3'
AtBiP3SP-GFP-F	5'-GAAGAGCAGAAACTGGGGACGATGGGCAAAGGAGAAGAAC-3'
AtBIP3SP-F	5'-ATGATTTTTATCAAGGAAAACACAG-3'
AtBIP3-R	5'-CTATAACTCATCGTGATCATCTCC-3'
AtBiP1SP-GFP-F	5'-ATGGCTCGCTCGTTTGGAGCTAAC-3'
AtBiP1SP-GFP-R	5'-TTATTTGTATAGTTCATCCATGCC-3'
AtBiP2SP-GFP-F	5'-ATGGCTCGCTCGTTTGGAGCTAAC-3'
AtBiP2SP-GFP-R	5'-TTATTTGTATAGTTCATCCATGCC-3'
AtBiP3SP-GFP-F	5'-ATGATTTTTATCAAGGAAAACACAG-3'
AtBiP3SP-GFP-R	5'-TTATTTGTATAGTTCATCCATGCC-3'
AtBiP1 Fw	5'-CACCATGGCTCGCTCGTTTGGAGCT-3'
AtBiP1 Rv Non-Stop	5'-GAGCTCATCGTGAGACTCATCT-3'
AtBiP1 Rv Stop	5'-CTAGAGCTCATCGTGAGACTCA-3'
AtBiP2 Fw	5'-ATGGCTCGCTCGTTTGGAGCA-3'
AtBiP2 Fw	5'-TAATATGCGGCCGCATGGCTCGCTCGTTTGGA-3'
AtBiP2 Rv Non-Stop	5'-ATTAATGCGGCCGCGAGCTCATCGTGAGACTC -3'
AtBiP2 Rv Stop	5'-ATTAATGCGGCCGCGAGCTCATCGTGAGACTC-3'

AtBiP2 Rv Stop	5'-CTAGAGCTCATCGTGAGACTC -3'
AtBiP2 Rv Non-Stop	5'-GAGCTCATCGTGAGACTCATC-3'
AtBiP1 Fw	5'-CACCATGGCTCGCTCGTTTGGAGCT-3'
AtBiP3 Fw	5'-CACCATGATTTTTATCAAGGAAAACACA-3'
AtBiP3 Fw	5'-ATGATTTTTATCAAGGAAAAC-3'
AtBiP3 Rv Stop	5'-CTATAACTCATCGTGATCATCTCCA-3'
AtBiP3 Rv Non-Stop	5'-TAACTCATCGTGATCATCTCCA-3'
AtERdj2A Fw	5'-CACC-ATGGCGGCGTCAGAAGAGAATA-3'
AtERdj2A Fw	5'-ATGGCGGCGTCAGAAGAGAATA-3'
AtERdj2A Nonstop Rv	5'-CTCTTCCTCCGATCCCGACTCT-3'
AtERdj2A Stop Rv	5'-TCACTCTTCCTCCGATCCCGAC-3'
AtERdj2A Rv	5'-ATTAATGCGGCCGCGG-CTCTTCCTCCGATCCCGA-3'
KpnI-Prom ERdj2A Fw	5'-AGAATTGGTACCCCATGGCGGCGTCAGAAGAGAA-3'
NotI-Prom ERdj2A Fw	5'-ACAGCGGCGGCCGC-GCGTAAAGCTCTTCCGTCC-3'
Prom ERdj2A Fw	5'-GCGTAAAGCTCTTCCGTCCAA-3'
DnaJ Domain Fw	5'-ATGGACCCATTCAGTATACTTGG-3'
DnaJ Domain Rv	5'-CTAACCATACTTCTCAAAGTTTTCA-3'
C-Domain Fw	5'-ATGCTGGCCCCAAGCAAAGTTAT-3'
C-Domain Rv	5'-ATGCTGGCCCCAAGCAAAGTTAT-3'
AtERdj3A Fw	5'-CACC-ATGGTGAGAACAAGATTGG-3'
AtERdj3A Rv Nonstop	5'-TTTGATCTGGGGTTTCTGTCTT-3'
AtERdj3A Rv Stop	5'-TCATTTGATCTGGGGTTTCTGT-3'
CMV-Fny CP-F	5'-GGGAGTGAACGCTGTAGACCTGG-3'
CMV-Fny CP-R	5'-AGAGATGGCGGCAACGGATA-3'
CMV-Q CP-F	5’-TGTTTTCTTTGTTTTGCGTCTCAGT-3'
CMV-Q CP-R	5'-TTTCAGGCGGTTTCAGGGTAA-3'
*erdj2a-1*LP	5’-TTATGTGATCGCAGGGATTTC-3’
*erdj2a-1*RP	5’-CTGTAAGAAAGGGGAAATGCC-3’
*erdj2b-1*LP	5’-AATCTCTGCGTATTCAGCTGC-3’
*erdj2b-1*RP	5’-GAGTGTGATCGTTCAGGGAAG-3’
*erdj2b-2*LP	5’-CTCCTTCACGTTTGTTCTGATG-3’
*erdj2b-2*RP	5’-TATCATTTGGAGACGGCAAAG-3’

*erdj3a-1*LP	5'-AATCTCGATTTTGTATGGGCC-3'
*erdj3a-1*RP	5'-TTCACGCTACAGAGTAAAGTCCC-3'
bip1-4(*bip1*)LP	5'-ATTAACAGGTTTGGTGCTCTTTTC-3'
bip1-4(*bip1*)RP	5'-AACCAAGAAATCTCAGGTTTTCAC-3'
P745	5'-AACGTCCGCAATGTGTTATTAAGTTGTC-3'
bip2-1(*bip2*)LP	5'-CGACAGGTGCGACTAAAAATC-3'
bip2-1(*bip2*)RP	5'-AGGTCACATTTGAAGTGGACG-3'
LB1	5'-GCCTTTTCAGAAATGGATAAATAGCCTTGCTTCC-3'
LB2	5'-GCTTCCTATTATATCTTCCCAAATTACCAATACA-3'
LB3	5'-TAGCATCTGAATTTCATAACCAATCTCGATACAC-3'
bip2-2(*bip2w*)LP	5'-CGACAGGTGCGACTAAAAATC-3'
bip2-2(*bip2w*)RP	5'-AGGTCACATTTGAAGTGGACG-3'
bip3-1(*bip3*)LP	5'-TGGTGAAGGTGGAGAAGAAAC-3'
bip3-1(*bip3*)RP	5'-GCCATGCCATAGAAAAGTTCC-3'
Salk-LBb1.3	5'-ATTTTGCCGATTTCGGAAC-3'
AtActin-F	5'-GGCCGTTCTTTCTCTCTATGC-3'
AtActin-R	5'-CCCTCGTAGATTGGCACAGT-3'
AtUBQ1-F	5'-AGAGCTGTCAACTGCAGGAAGAA-3'
AtUBQ1-R	5'-ACAAGAAAAACAAACCCTATCAAAGG-3'
AtEF1α-F	5'-GCCTGGTATGGTTGTGACCT-3'
AtEF1α-R	5'-GAAGTTAGCAGCACCCTTGG-3'

Table S2. List of identified interacting proteins with CMV MP in Arabidopsis PECP. PECP proteins were co-immunoprecipitated with CMV MP-4M8H using anti-c-Myc antibody. Proteins were separated by SDS-PAGE, and individual bands were excised from the gel for LC-MS/MS analysis. Three biological replicates were analyzed to identify PECP proteins which interact with CMV MP. MS spectra were matched to peptides with the false discovery rate (FDR) of ≤ 0.01. Identified proteins were detected within at least two of three biological replicates.

Accession	Annotation	Matched peptide count	Unique peptide count	Molecular weight (kDa)	Sequence coverage (%)
AT5G24010	Protein kinase family protein	11	1	92	4
AT1G79940	AtERdj2A	3	1	77	2.8
AT4G21180	AtERdj2B	3	1	75	2
At5g28540 (At5G42020)	BiP1/BiP2, Luminal binding proteins	6	2	74	6
AT1G52400	β-glucosidase 1	3	1	59	5
AT5G20630	Germin-like protein 3	7	5	22	23
AT5G42980	Thioredoxin H-type 3	3	1	13	15
